# NDUFA4L2 Regulated by HIF-1α Promotes Metastasis and Epithelial–Mesenchymal Transition of Osteosarcoma Cells Through Inhibiting ROS Production

**DOI:** 10.3389/fcell.2020.515051

**Published:** 2020-11-20

**Authors:** Wen-Ning Xu, Run-Ze Yang, Huo-Liang Zheng, Lei-Sheng Jiang, Sheng-Dan Jiang

**Affiliations:** Department of Clinic of Spine Center, Xinhua Hospital, Shanghai Jiao Tong University School of Medicine, Shanghai, China

**Keywords:** HIF-1a, hypoxia, osteosarcoma, metastases, NDUFA4L2

## Abstract

Osteosarcoma (OS) accounts for a large proportion of the types of bone tumors that are newly diagnosed, and is a relatively common bone tumor. However, there are still no effective treatments for this affliction. One interesting avenue is related to the mitochondrial NDUFA4L2 protein, which is encoded by the nuclear gene and is known to be a critical mediator in the regulation of cell survival. Thus, in this study, we aimed to investigate the effect of NDUFA4L2 upon the metastasis and epithelial–mesenchymal transition of OS. We found that NDUFA4L2 protein expression was upregulated in hypoxic conditions. We also used 2-ME and DMOG, which are HIF-1α inhibitors and agonists, respectively, to assess the effects related to decreasing or increasing HIF-1α expression. 2-ME caused a significant decrease of NDUFA4L2 expression and DMOG had the opposite effect. It was obvious that down-regulation of NDUFA4L2 had a direct interaction with the apoptosis of OS cells. Western blotting, wound healing analyses, Transwell invasion assays, and colony formation assays all indicated and supported the conclusion that NDUFA4L2 promoted OS cell migration, invasion, proliferation, and the epithelial–mesenchymal transition. During experiments, we incidentally discovered that autophagy and the ROS inhibitor could be used to facilitate the rescuing of tumor cells whose NDUFA4L2 was knocked down. Our findings will help to further elucidate the dynamics underlying the mechanism of OS cells and have provided a novel therapeutic target for the treatment of OS.

## Introduction

Osteosarcoma (OS) accounts for a large proportion of primary malignant bone tumors that significantly affect children, teenagers, and young adults, accounting for 20–35% of all such diagnoses (Torre et al., 2015). Although there are many treatments that have emerged in recent years, such as chemotherapy and effective resection, the 5-year overall survival rate of OS remains poor, mainly as a result of metastases and relapse (Harrison et al., 2018). The underlying mechanisms inducing the evolution and progression of OS remain poorly understood. Consequently, it is of significance for the research community to elucidate the potential mechanisms of OS and to facilitate the discovery of novel and effective treatment approaches.

The oxygen concentration of normal non-diseased tissue is about 4%, while the oxygen concentration of solid tumorous tissues is <1% (Muz et al., 2015). OS cells usually survive in low-oxygen conditions, and this advantage plays an important role in the rapid rates of tumor proliferation (Ebbesen et al., 2004). Cells of solid tumors that have survived in a hypoxic environment can activate hypoxic gene pathways and accelerate tumor chemoresistance, which eventually causes a poor prognosis (Keith and Simon, 2007). Hypoxia inducible factor-1 (HIF-1) protein can regulate hypoxic genes of cancer cells and thus can help cope with the hypoxic environments. Notably, HIF-1 protein is comprised of a hypoxia-regulated α subunit and a non-hypoxia-regulated β subunit (Goda et al., 2003). In a normoxic environment, HIF-1α is hydroxylated by prolyl hydroxylases, and hydroxylated HIF-1α is eventually disintegrated by the proteasome. Activated prolyl hydroxylases can be inhibited when cancer cells are exposed to hypoxic environments and when they become hypoxic (Huang et al., 1998; Maxwell et al., 1999; Jaakkola et al., 2001).

NDUFA4L2, a component of the electron transport chain (ETC) complex I subunit, is highly expressed in hypoxic environments, and plays an important role in fine adjustments of complex I activity. Consequently, NDUFA4L2 can mediate the function of oxidative phosphorylation and reactive oxygen species (ROS) production in mitochondria. At present, little is known with respect to the function of NDUFA4L2, especially in regards to its possible functions and roles in OS development. NDUFA4L2 was knocked out in cells, which survived in a hypoxic environment and subsequently promoted mitochondrial ROS production (Tello et al., 2011). This indicated that the NDUFA4L2 protein repressed ROS production and consequently induced anti-oxidative stress in cancer cells (Tello et al., 2011). DNA destruction induced by high ROS accumulation is also known to have detrimental effects upon the survival, proliferation, and metastasis of cancer cells. A recent study reported that NDUFA4L2 accelerated the survival of Non-small cell lung cancer (NSCLC) in hypoxic conditions (Meng et al., 2019). However, the underlying mechanisms of how NDUFA4L2 appears to control and influence the survival of OS is unknown.

Autophagy, a highly conserved biological process, disposes of abnormal or misfolded proteins and limits dysregulation as well as unnecessary organelles in a lysosome-dependent manner (Weckman et al., 2015; Mowers et al., 2017). In recent decades it has emerged that dysfunction of autophagy induced the pathogenesis of varied types of neural diseases (Umezawa et al., 2018), metabolic defects, and the onset of tumors (Huang et al., 2017). However, the specifics of interactions between autophagy and NDUFA4L2 in OS are currently unknown. Therefore, in our study, we sought to explore the influence of NDUFA4L2 and autophagy to the pathogenesis of OS.

## Materials and Methods

### Cell Lines and Cell Culture

The human OS cell lines 143b, HOS, and U2OS were purchased from ATCC Company (Manassas, VA), and were cultured in DMEM modified medium (Gibco, Invitrogen). All medium contained 10% fetal bovine serum, 100 units/mL penicillin and 100 μg/mL streptomycin. Cells were cultured in a consistent and humidified atmosphere of 5% CO_2_ at 37°C. Cells were cultured in hypoxic environments (1% O_2_) until 70% density.

### Cell Proliferation Assay

We used the Cell Counting Kit-8 (CCK-8; Beyotime, Beijing, China) to measure cell viability. 143b, HOS, and U2OS cells were adjusted to 1 × 10^5^ cells/well and were seeded in six-well plates. 24, 48, 72 and 96 h post-transfection, 20 μL of CCK-8 solution was added per well. After 4 h of incubation, cell proliferation was determined by measuring optical density (OD) values at 450 nm on a Microplate Reader (Thermo Fisher Scientific).

### Colony Formation Assays

Colony formation assays were performed following previously published methods (Cai et al., 2017). Transfected cells during logarithmic phases were plated in six-well plates at a density of 1,000 cells per well. Two weeks later, we twice washed the cells by using phosphate buffered saline (PBS), and then fixed the samples by using methanol for 30 min. Colonies were then stained by using 0.1% crystal violet (Sinopharm Chemical Reagent, Shanghai, China), and the numbers of colonies were manually calculated.

### Flow Cytometry to Detect Cell Apoptosis

Flow cytometry was used to measure the rate of apoptosis by using the Annexin V-FITC Apoptosis Detection Kits (BD Biosciences, Franklin Lakers, NJ) according to the manufacturer’s protocol. Cells were incubated with the mixing solution at room temperature for 15 min, and the cells were analyzed by using the FACS System (BD Biosciences).

### Immunofluorescence

Cells were seeded on glass coverslips and then fixed with 4% paraformaldehyde for 30 min. The cells were permeabilized in 0.1% Triton X-100 for 20 min. Next, cells were incubated with primary antibodies overnight at 4•C as follows: E-cadherin (1:100, Abcam, United States) and Vimentin (1:200, Abcam, United States). Coverslips were thrice washed with PBST after they were incubated with secondary antibody for 1.5 h at 37•C (Beyotime, China). Immunofluorescence was captured through photography and by using fluorescence microscopy (Olympus BX51).

### Western Blotting Assays

The treated cells were harvested and lysed using RIPA buffer (with protease inhibitors). Nuclear protein was harvested by using Nuclear protein and cytoplasmic protein extraction kit (Beyotime, China). Cell lysates were separated on SDS-PAGE, transferred onto PVDF membranes, blocked with 5% BSA, incubated with primary antibodies against the following: HIF-1α (1:1,000, Proteintech, China), HIF-2α (1:1,000, abcam, United Kingdom), NDUFA4L2 (1:1,000, Proteintech, China), Cytochrome c (1:1,000, Proteintech, China), P62 (1:1,000, Proteintech, China), Beclin-1 (1:1,000, CST, United States), LC3(1:1,000, Novus, United States), Bax (1:1,000, Proteintech, China), Bcl-2 (1:1,000, Proteintech, China), PARP (1:1,000, CST, United States), E-cadherin (1:1,000, CST, United States), Vimentin (1:1,000, CST, United States), Slug (1:1,000, CST, United States), Snail (1:1,000, CST, United States), MMP2 (1:1,000, CST, United States), MMP9 (1:1,000, CST, United States), Lamin B (1:1,000, Beyotime, China), Tubulin (1:1,000, Beyotime, China) and GAPDH (1:1,000, Beyotime, China). Subsequently, membranes were incubated with secondary antibodies and measures were determined using EasyBlot ECL kits (Sangong, Songjiang, Shanghai, China). Membranes were thrice washed with TBST and were then incubated with HRP-conjugated secondary antibodies. Finally, membranes were induced to react with the addition of ECL Plus reagent (Millipore). Results were quantified by Image-J (National Institutes of Health).

### RNAi, pcNDA, and Lentivirus Transfection

NDUFA4L2-siRNAs and respective negative control (NC) siRNAs were designed, synthesized, and purchased from GenePharma, China. siRNAs sequences are listed as follows: 5′-CUGAUGACCAGCAACUUGAdTdT-3′ (sense); 5′-UCAAGUUGCUGGUCAUCAGdTdT-3′ (antisense) for si-HIF-1α-1; 5′-GGGCCGUUCAAUUUAUGAATT-3′ (sense) and 5′-GCCUCUUCGACAAVCUUAATT-3′ (antisense) for si-HIF-1α-2; 5′-CAGCAUCUUUGAUAGCAGUdTdT-3′ (sense) and 5′-ACUGCUAUCAAAGAUGCUGdTdT-3′ (Antisense) for si-HIF-2α-1; 5′-CACCGCCGTACTGTCAACCTCAAGTTTCAAGAGAACTT GAGGTTGACAGTACGGCTTTTTTG-3′ (sense) and 5′-GATCCAAAAAAGCCGTACTGTCAACCTCAAGTTCTCTT GAAACTTGAGGTTGACAGTACGGC-3′ (Antisense) for si-HIF-2α-2; 5′-UCCUCGGUACGUGUCACGUTT-3′ (sense) and 5′-ACGUGCCACGAUCGCAGAUTT-3′ (antisense) for si-NC; 5′-GCAGUUUCCACUGACUAUATT-3′ (sense) and 5′-UAUAGUCAGUGGAAACUGCTT-3′ (anti-sense) for si-NDUFA4L2-1; 5′-UCAUCCCGAUGAUCGGCUUTT-3′ (sense) and 5′-AAGCCGAUCAUCGGGAUGATT-3′ (anti-sense) for si-NDUFA4L2–2; 5′-GCUGGGACAGAAAGAACAATT-3′ (sense) and 5′-UUGUUCUUUCUGUCCCAGCTT-3′ (anti-sense) for si-NDUFA4L2–3. pcDNA-NDUFA4L2 plasmids were designed and synthesized chemically (Sangon Biotech, China). The cloning vector was pcDNA3.1+. As described above, NDUFA4L2 plasmids were added to serum-free medium, and then, Lipofectamine 2000 (Invitrogen, Carlsbad, CA) was added to the same medium. The Lentivirus containing shNDUFA4L2 or shNC were transfected into OS cells. After 48 h, cells were used in follow-up experiments.

### Chromatin Immunoprecipitation (ChIP) Assays

We used SimpleChIP Enzymatic Chromatin IP kit (Magnetic Beads; CST, Pudong, Shanghai, China) to conduct the ChIP assays following all manufacturer protocols. The precipitated protein/DNA complexes were separately immunoprecipitated with the use of antiSP1 antibody (Abcam, Pudong, Shanghai, China) and IgG antibody (Abcam, Pudong, Shanghai, China). The precipitated DNA was then analyzed in quantitative real time polymerase chain reaction (qRT-PCR). Primer Sequences for CHIP Assays were as follows: Forward: 5′–CAGGTCTGTGTATGTGTGAAA–3′, and Reverse: 5′–CTACGCACTGTCACTGAG–3′.

### Transwell Assays

Transwell invasion assays were performed to assess cell invasion. Upper chambers were coated with Matrigel (Corning, NY) and then incubated overnight before cells were plated. Transfected cells were cultured in upper chambers with serum-free medium. In the lower chambers, DMEM with 10% FBS was added. Post 24 h of incubation, remaining non-invaded cells were carefully wiped away. Finally, invaded cells were stained with 0.1% crystal violet (Sinopharm Chemical Reagent). Invaded cells were counted under light microscopy (Olympus, Tokyo, Japan).

### Migration Assays

To measure cell migratory ability, 143b were seeded onto a 6 cm plate overnight in a consistent and humidified atmosphere of 5% CO_2_ at 37°C. Confluent monolayers were scratched using sterile pipette tips followed by several washes with phosphate-buffered saline (PBS) to remove detached cells. Cells were then transfected with siRNA for 48 h in medium without serum. Photographs of wounded areas were obtained using a Leica DMI3000 B inverted microscope (Leica Microsystems GmbH, Wetzlar, Germany). The migration rate was calculated according to the scratched surfaces, which were quantified using ImageJ Version 1.410 software (National Institutes of Health, Bethesda, MD, United States).

### Tumor Xenograft Model

Immunodeficient male BALB/C nude mice (18–20 g) were obtained from the Animal Center of Shanghai Jiao Tong University. Animals were cared for at least 1 week before initiation of experimental phases. Animals were fed with rodent laboratory chow and water *ad libitum* under standard laboratory animal conditions (25°C, 50–70% humidity, 12 h light/dark cycle). Each nude mouse (Five mice per treatment group) was injected subcutaneously with 143b cells (100 μ, 1 × 10^6^) transfected with LV-shNC or LV-shNDUFA4L2. After 2 weeks, mice were sacrificed and tumors were excised. Tumors were weighed as well as subjected subsequently to immunohistochemical assays. All experiments were carried out in accordance with Guidelines for the Care and Use of Laboratory Animals and approved by the Xinhua Hospital, Shanghai Jiao Tong University School of Medicine.

### Immunohistochemical Examination

Immunohistochemical examinations were performed following methods outlined in a previous study. Briefly, antigen was retrieved and microwaved for 15 min. Next, endogenous peroxidase activity was blocked for 10 min by use of 3% hydrogen peroxide, and then non-specific binding sites were blocked for 30 min at room temperature by 5% BSA (bovine serum albumin). Primary antibodies were added to sections and incubated overnight at 4•C. For primary antibodies we used anti-LC3, 1:100, Novus, United States, and (PCNA, 1:100, Proteintech, China). Sections were incubated with an appropriate HRP-conjugated secondary antibody (Santa Cruz Biotechnology, Dallas, TX, United States) and counterstained with hematoxylin.

### ROS Measurement

Mitochondrial ROS production was detected by Reactive Oxygen Species Assay Kit (Beyotime, China) according to the manufacturer’s protocol. After various treatments, Os cells were washed with PBS and incubated with serum-free medium containing with DCFH-DA at 37°C for 20 min. Then DCFH-DA was removed and washed with serum-free medium three times. DCF fluorescence distribution of cells was detected using fluorescence microscope analysis (Olympus Fluoview, Japan). Positive cells were emitted with green.

### TUNEL Assays for Apoptosis

OS cell apoptosis was determined using One-step TUNEL cell apoptosis detection kits (Beyotime, No. C1086, Shanghai, China) following manufacturer protocols. OS cells were seeded upon coverslips in six-well plates. Post-application of varied treatments, cells were washed using PBS. Cells were then fixed with 4% paraformaldehyde for 30 min and then washed once with PBS. Cells were permeabilized with PBS containing 0.3% Triton X-100 at room temperature for 5 min. Cells were then twice washed with PBS. We then added 50 μL of TUNEL detection solution to samples and incubated at 37°C for 60 min in the dark. Finally, we added DAPI, incubated for 5 min, and completed imaging using fluorescence microscopy (Olympus BX51).

### Oxygen Consumption

Oxygen consumption (OCR) was measured by high-resolution respirometry (Oxygraph-2k, Orobros Instruments, Innsbruck, Austria). After OS cells were trypsinized, they were resuspended in HBSS containing 25 mM HEPES at 2 × 10^6^ cells/ml. The instrument background flux was calculated as a linear function of oxygen concentration, and the experimental data were corrected using DatLab software (Oroboros Instruments). The oxygen concentration in the air saturated medium was 175.7 μM at 37°C. The oxygen concentration of cells was measured in a 37°C box under normoxic and hypoxic (1% O2) environments with the indicated treatments.

### Lactate Production

To evaluate the production of lactate, a lactic acid assay kit (BioVision) was used to explore the cell culture medium according to the manufacturer’s protocol. These values were normalized to protein concentration.

### Statistical Analyses

Statistical analyses were performed using GraphPad Prism 5. All data were presented as the mean ± standard deviation (SD). Independent-sample *t*-tests were performed to facilitate analysis of differences between treatments. For multiple comparisons, a two-way analysis of variance (ANOVA) was performed followed by a Tukey *post-hoc* test. All p-values were two-sided and nsp ≥ 0.05, ^∗^*p* < 0.05, ψ*p* < 0.01, and #*p* < 0.001 were deemed as levels of statistical significance. All experiments were replicated three times.

## Results

### HIF-1α Can Regulate NDUFA4L2 Expression in 143b and U2OS in Hypoxic Conditions

**FIGURE 1:**
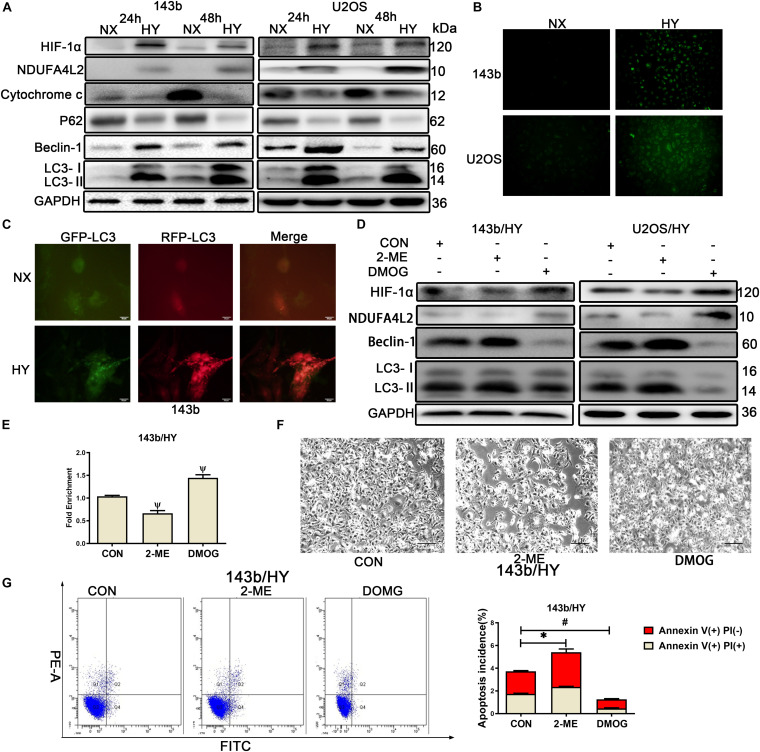
HIF-1α might regulate the expression of NDUA4L2 in 143b and U2OS cell lines. 143b and U2OS cells were cultured in hypoxic environments for 24 and 48 h while control cells were cultured in normoxic environments. **(A)** Protein expression of HIF-1α, NDUFA4L2, P62, Beclin-2, LC3, and GAPDH in 143b and U2OS cells was determined by Western blotting. **(B)** ROS production was detected in 143b and U2OS under normoxic environments or hypoxic environments for 48 h by use of a Reactive Oxygen Detection Kit. **(C)** Fluorescence-based imaging for 143b cells transfected with mRFP-GFP-LC3 adenovirus. Green dot represents the start of autophagic flux, and Red dot represents the end of autophagic flux. Promotion of Red dot represents that autophagic flux was promoted. 143b cells were under normoxic environments or hypoxic environments for 48 h. **(D)** Expression of HIF-1α, NDUFA4L2, Beclin-1, LC3, and GAPDH were determined by Western blotting in 143b and U2OS cells pretreated with 2-ME and DMOG in hypoxic environments. **(E)** CHIP assay of HIF-1α binding to NDUFA4L2 gene in 143b cells. **(F)** About 1 × 10^6^ 143b cells were planted into six-well plates and cultured at 37•C until 70% confluence. 143b cell morphology was photographed using microscopy. Scale Bar = 100 μm. **(G)** Apoptosis of 143b cells was detected by flow cytometry. NX, normoxic environment; HY, hypoxic environment. nsp ≥ 0.05, ^∗^*p* < 0.05, ψ < 0.01, and # < 0.001 were defined as measures that indicated significant differences among treatment groups. All experiments were performed in triplicate.

To investigate hypoxia adaptation mechanisms in human OS, we performed Western blotting and characterized levels of gene expression of two human OS cell lines, 143b and U2OS. Cells were cultured in normoxic and hypoxic environments for 24 and 48 h, respectively. The results for 143b and U2OS indicated that HIF-1α and NDUFA4L2 expression was significantly upregulated under hypoxic conditions (Figures 1A,B). We further examined the Cytochrome C and autophagy relative protein, p62, Beclin-1, LC3, and found that the activity of autophagy was much higher under hypoxic vs. non-hypoxic conditions (Figure 1A). In hypoxic environments, Cytochrome c was released to cytoplasm from mitochondria, indicating that there was some apoptosis in OS cells cultured in hypoxic environments (Supplementary Figure S1A). The following experiments were performed in hypoxic environments for 48 h. The ROS production was also increased in hypoxic environments (Figure 1B). The lower OCR and higher lactate production demonstrated that OS cells mainly used glycolysis to produce energy in hypoxic environments (Supplementary Figures S1B,C). An autophagy indicator was used to track autophagy flux, and results indicated that autophagy flux was significantly enhanced under hypoxic environments (Figure 1C). Thus, we inferred there were important connections among NDUFA4L2, autophagy, and apoptosis.

Treatment of cells with HIF-1α inhibitor (2-methoxyestradiol, 2-ME, 20 mmol/L) (Chen et al., 2015) and agonist (Dimethyloxaloylglycine, DMOG, hydroxylase inhibitor, 0.2 mmol/L) (Xie et al., 2012), respectively was performed. Western blotting results revealed that suppression of HIF-1α induced reductions in NDUFA4L2 and that accelerated HIF-1α expression could heighten NDUFA4L2 protein expression compared to the control group (Figure 1D). CHIP assays were performed on 143b cells. To determine the level of consequence that HIF-1α bound to NDUFA4L2’ HREs, qPCR was carried out with specific primer for attachment to the HRE site. Schematic representations of the human NDUFA4L2 gene and the nucleotide sequences matching HRE from six mammalian genes was provided in Supplementary Figure S1D. The level of consequence that HIF-1αbound to NDUFA4L2’ HREs was found to have decreased in the 2-ME treatment group and contrastingly increased in the DMOG treatment group (Figure 1E). 143b cells that were treated with HIF-1α agonist were found to have had vigorous growth compared to the control group as characterized under light microscopy. However, we found opposite results in the treatment of 143b cells with 2-ME (Figure 1F). The cell proliferation detected by CCK-8 confirmed that HIF-1(α inhibitor 2-ME repressed the cell proliferation of OS cells and HIF-1(α agonist DMOG promoted the cell proliferation of OS cells in hypoxic environments (Supplementary Figure S1E). Importantly, the incidence of apoptosis was detected by use of flow cytometry Annexin V/PI double staining. Results confirmed that 143b cells treated with DMOG had lower apoptosis incidence (Figure 1G). However, the very low percentages of dead cells seemed to represent a normal rate of cell death rather than actually increased apoptosis. Therefore, we pretreated the cells with staurosporine (100 nM) (Lien et al., 2018) to quantify the relative effect of DMOG and ME treatments upon dead cells. The results confirmed the above findings that OS cells treated with DMOG had a lower incidence of apoptosis (Supplementary Figure S1F). We further examined autophagy relative proteins, Beclin-1 and LC3. We found that autophagy activity was significantly inhibited when DMOG was added to 143b and U20S, and autophagy activity was significantly promoted when 2-ME was added to the OS cell lines (Figure 1D).

To further confirm the relationship between HIF-1α and NDUFA4L2, small interfering RNAs to HIF-1α and HIF-2α were established to facilitate silencing of expression HIF-1α and HIF-2α. The effect of si-HIF-1α and si-HIF-2α in hypoxic environments was confirmed by use of qRT-PCR and Western blotting. HIF-1α protein expression was knocked down by si-HIF-1(α-1 and si-HIF-1(-2 significantly (Supplementary Figure S1G). HIF-1α knockdown induced decreased expression of NDUFA4L2 protein in OS cells (Supplementary Figure S1G). CHIP assays indicated that HIF-1α bound to NDUFA4L2’ HREs was decreased by way of si-HIF-1α (Supplementary Figure S1I). Moreover, we found that HIF-2α knockdown by si-HIF-2α-1 and si-HIF-2α-2 did not induce a reduction in expression of NDUFA4L2 and HIF-2α bound to NDUFA4L2’ HREs did not decreased by way of si-HIF-2α (Supplementary Figures S1H,J). These results revealed that HIF-1α regulated expression of NDUFA4L2 and regulated levels of autophagy in hypoxic environments.

### Knockdown of NDUFA4L2 Inhibits Osteosarcoma Cell Proliferation, Migration, and Epithelial–Mesenchymal Transition Progression and Promotes Apoptosis *in vitro*

**FIGURE 2:**
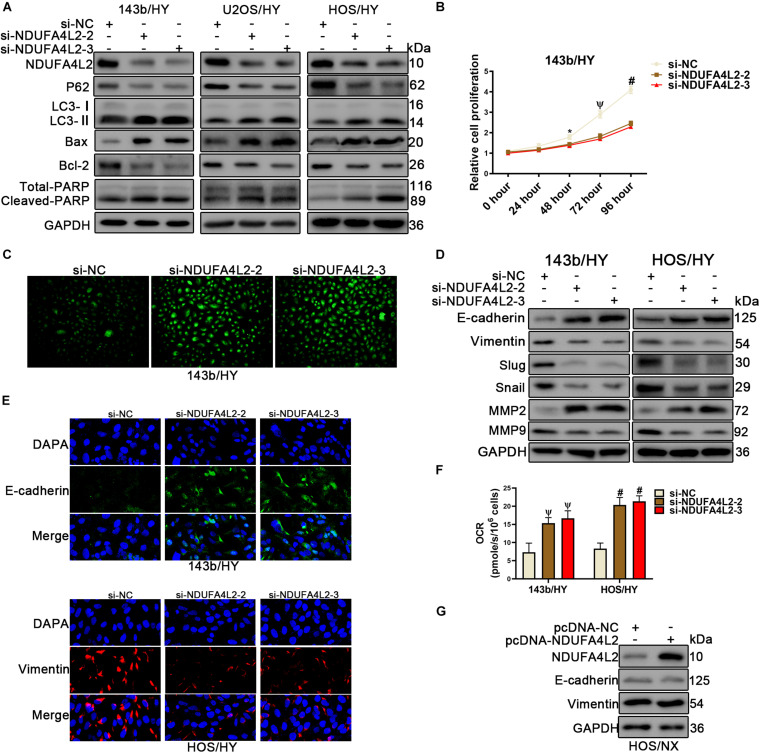
Knockdown of NDUFA4L2 inhibited OS cell proliferation migration, and epithelial–mesenchymal transition progression, as well as promotes apoptosis *in vitro*. 143b, U2OS, and HOS cell lines were transfected with si-NC or si-NDUFA4L2-2 or si-NDUFA4L2-3 in hypoxic environments. **(A)** Protein expression of NDUA4L2, P62, LC3, Bax, Bcl-2, PARP, and GAPDH in 143b, U2OS, and HOS cells was measured using Western blotting. **(B)** Relative cell proliferation of 143b cells was detected by CCK-8. **(C)** ROS production was detected by use of a Reactive Oxygen Detection Kit. **(D)** Protein expression of Slug, snail, MMP2, MMP9, E-cadherin, and Vimentin was measured in 143b and HOS cells post-transfection of si-NC, si-NDUFA4L2-1, or si-NDUFA4L2-2. **(E)** Immunofluorescence assessments were performed to measure E-cadherin and Vimentin protein expression in 143b and HOS cells. **(F)** Protein expression of NDUFA4L2, E-cadherin, and Vimentin was measured in HOS cells. NX, normoxic environment; HY, hypoxic environment. nsp ≥ 0.05, *p < 0.05, ψ < 0.01, and # < 0.001 were defined as measures that indicated significant differences among treatment groups. All experiments were performed in triplicate. **(F)** OCR were detected in 143b and HOS cells. **(G)** Protein expression of NDUFA4L2, E-cadhrin, and Vimentin was measured in HOS cells.

We found that NDUFA4L2 expression was increased, but also found that Cytochrome c expression was decreased in mitochondria of OS cells under hypoxic environments. Consequently, to examine if NDUFA4L2 could inhibit apoptosis in OS cell lines cultured in hypoxic environments, small interference including si-NC, si-NDUFA4L2-1, si-NDUFA4L2-2 and si-NDUFA4L2-3 was used to knock down NDUFA4L2 in 143b, U2OS, and HOS cells cultured under hypoxic environments. Western bolting results showed that NDUFA4L2 was silenced significantly by si-NDUFA4L2-1, si-NDUFA4L2-2, and si-NDUFA4L2-3 (Supplementary Figure S2A). Bcl-2/Bax has a negative correlation with apoptosis incidence. The more apoptosis happens, the greater ratio of C-PARP/T-PARP is observed in apoptosis cells. Figure 2A demonstrated that knockdown of NDUFA4L2 induced decreased expression of Bcl-2/Bax and increased expression of C-PARP/T-PARP compared to the control group in 143b, U2OS, and HOS cell lines. Flow cytometry Annexin V/PI double staining and TUNEL assays demonstrated that si-NDUFA4L2 promoted apoptosis of HOS and 143b cells (Supplementary Figure S2C). At the cellular level, we used CCK-8 to examine relative cell proliferation whereby results suggested that proliferation was reduced in cells whose NDUFA4L2 was knocked down (Figure 2B and Supplementary Figure S2B). Furthermore, silencing of NDUFA4L2 induced production of ROS (Figure 2C). Intriguingly, autophagy was increased in these cells transfected with si-NDUFA4L2 (Figure 2A). We inferred that mitochondrial NDUFA4L2 might be essential for OS cells survival. However, the role of autophagy still remains unknown and was elucidated in our study.

To assess if NDUFA4L2 regulated cancer metastasis in OS cells, we established small interference RNA to silence NDUFA4L2. The results of associated wound healing assays suggested that 143b and HOS cells transfected with si-NDUFA4L2 had reduced migration related abilities (Supplementary Figure S2F). At the molecular level, results of immunofluorescence and Western blotting indicated that there were increases in epithelial–mesenchymal transition (EMT) relative protein E-cadherin expression, and decreases in EMT relative protein Vimentin in OS cells transfected with si-NDUFA4L2 (Figure 2E and Supplementary Figure S2E). Silencing of NDUFA4L2 also induced an increase in measures of E-cadherin, and induced a decrease in Vimentin, Slug, Snail, and MMP9 expression (Figure 2D). However, MMP2 protein expression was increased in cells transfected with si-NDUFA4L2. Importantly, NDUFA4L2 knockdown led to an increased OCR in OS cells under hypoxic environments (Figure 2F). Finally, we established pcDNA-NDUFA4L2 to overexpress NDUFA4L2 expression in HOS cells cultured in normoxic environments. Our results showed that overexpression of NDUFA4L2 in normoxia was insufficient alone to activate the EMT progression (Figure 2G). These results revealed that mitochondrial NDUFA4L promoted OS cell migration and EMT progression in hypoxic environments.

### NDUFA4L2 Protects Osteosarcoma Cells by Repressing ROS Production

**FIGURE 3:**
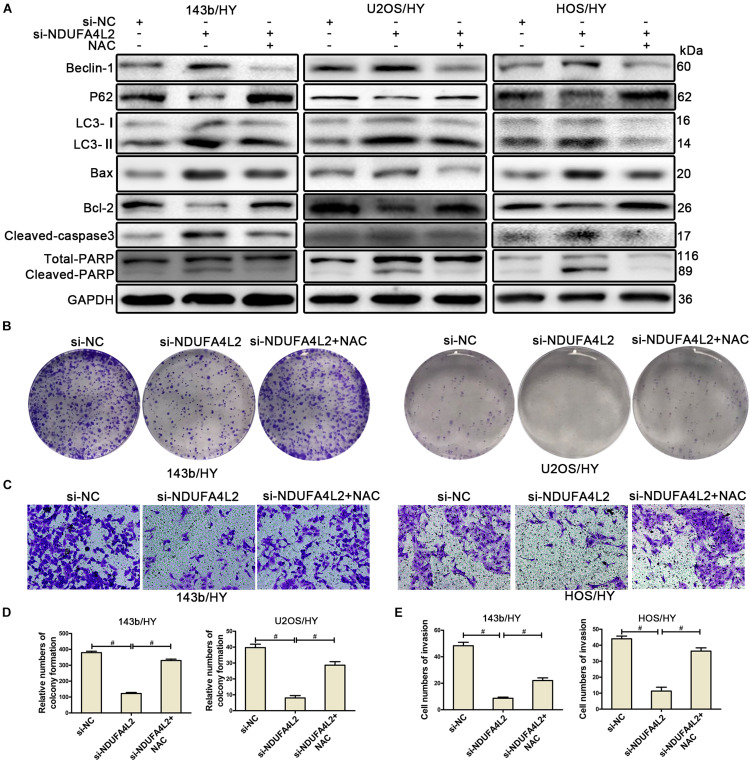
NDUFA4L2 protected OS cells by repressing ROS production. 143b, U2OS, and HOS cells were treated with NAC post-transfection with si-NC, si-NDUFA4L2-1, or si-NDUFA4L2-2 in hypoxic environments. **(A)** Western blotting facilitated determinations of protein expression of Beclin-1, P62, LC3, Bax, Bcl-2, Cleaved-caspase3, PARP, and GAPDH in 143b, U2OS, and HOS cells. **(B,D)** The colonizing ability of si-NDUFA4L2-transfected 143b and U2OS cells post-treatment with NAC was determined by using colony formation assays. **(C,E)** Invasion ability of si-NDUFA4L2-transfected 143b and HOS cells post-treatment with NAC was determined by using transwell assays. HY: hypoxic environment. nsp ≥ 0.05, and # < 0.001 were defined as measures that indicated significant differences among treatment groups. All experiments were performed in triplicate.

A recent study reported that NDUFA4L2 could function as an antioxidant (Lien et al., 2018). To confirm the role of ROS in OS cells post-silencing of NDUFA4L2, we applied NAC (N-acetylcysteine, 10mM) (Li et al., 2017), which is a scavenger of ROS, to treat 143b, U2OS, and HOS cells in hypoxic environments. NAC did not affect the control cells no matter whether or not OS cells were cultured in normal or hypoxic environments (Supplementary Figures S3A–C). NDUFA4L2 knockdown did not affect OS cells under normal environments either (Supplementary Figure S3C). NDUFA4L2 was downregulated in OS cells cultured in non-hypoxic environments. NDUFA4L2 knockdown did not induce an increase of ROS production and thereby NAC did not affect OS cells cultured in non-hypoxic environments. However, results of Western blotting indicated that NAC attenuated apoptosis and autophagy of OS cells transfected with si-NDUFA4L2 (Figure 3A). TUNEL assays confirmed that NAC rescued OS cell apoptosis (Supplementary Figure S3D). Ultimately, these results suggested that NAC could rescue the survival of OS cells under a hypoxic environment when NDUFA4L2 was knocked down.

To further investigate whether or not NDUFA4L2 regulated proliferation, invasion, and EMT progression of OS cell lines through the effect of repression of ROS production, we performed colony formation assays, transwell assays, and Western blotting to evaluate the proliferation, invasion, and EMT progression of OS cell lines transfected with si-NDUFA4L2 after treatment with the ROS inhibitor. Figures 3B,D indicated that 143b and U2OS cells transfected with si-NDUFA4L2 had a lower degree of colony formation than cells transfected with si-NC, whereas NAC could reverse this effect. We also observed that NAC promoted invasion of 143b and HOS cells transfected with si-NDUFA4L2. Results of transwell assays indicated that knockdown of NDUFA4L2 consequently induced inhibition of the invasion of 143b and HOS, whereas NAC could reverse this effect (Figures 3C,E). Furthermore, Western blotting indicated that NAC promoted EMT progression of OS cells transfected with si-NDUFA4L2 (Supplementary Figure S3E). These outcomes indicated that NDUFA4L2 could promote the proliferation, invasion and EMT progression of OS through repressing ROS production.

### Autophagy Promotes the EMT Progression, Invasion, Migration, and Proliferation in Osteosarcoma Cell Transfected With si-NDUFA4L2 by Eliminating ROS Production

**FIGURE 4:**
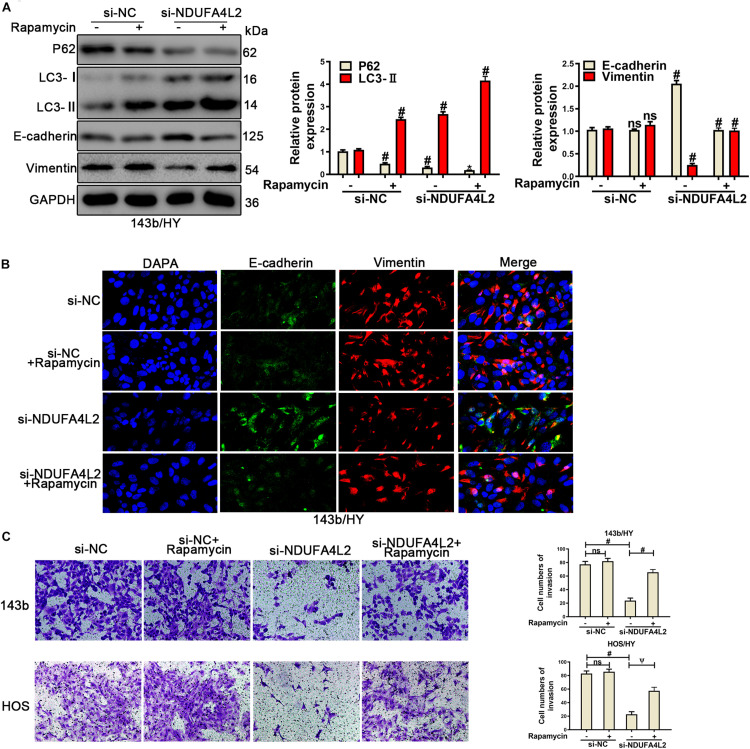
Upregulation of autophagy promoted the invasion and EMT progression of si-NDUFA4L2-transfected 143b and HOS cells. 143b and HOS cells were treated with Rapamycin post-transfection with si-NC or si-NDUFA4L2 in hypoxic environments. **(A)** Western blotting was performed to determine protein expression of P62, LC3, E-cadherin, and Vimentin in 143b cells. For P62, si-NC vs. si-NC + Rapamycin was #*p* < 0.001, si-NC vs. si-NDUFA4L2 was #*p* < 0.001, si-NDUFA4L2 vs. si-NDUFA4L2 + Rapamycin was ^∗^*p* < 0.05; For LC3, si-NC vs. si-NC + Rapamycin was #*p* < 0.001, si-NC vs. si-NDUFA4L2 was #*p* < 0.001, si-NDUFA4L2 vs. si-NDUFA4L2 + Rapamycin was #*p* < 0.001; For E-cadherin and Vimentin, si-NC vs. si-NC + Rapamycin was nsp ≥ 0.05, si-NC vs. si-NDUFA4L2 was # < 0.001, si-NDUFA4L2 vs. si-NDUFA4L2 + Rapamycin was # < 0.001. **(B)** Immunofluorescence was performed to measure E-cadherin and Vimentin expression in 143b cells. **(C)** Transwell assays were performed to determine the invasion ability of si-NDUFA4L2-transfected 143b and HOS cells post-treatment with Rapamycin. HY: hypoxic environment. nsp ≥ 0.05, ψ < 0.01, and # < 0.001 were defined as measures that indicated significant differences among treatment groups. All experiments were performed in triplicate.

According to previous research, we speculated that autophagy was positively correlated with metastasis of OS cells. Autophagy protected the survival, metastasis, and EMT progression by removing a large amount of ROS in OS cells when NDUFA4L2 was repressed. To confirm this role of autophagy in OS cells cultured in hypoxic environments, Rapamycin (10 nM) (Li et al., 2019) was used to treat 143b and HOS after transfection with si-NDUFA4L2. As demonstrated in Figure 4A, protein expression of P62 decreased and LC3 increased in si-NC + Rapamycin, si-NDUFA4L2 and si-NDUFA4L2 + Rapamycin based treatment groups. Compared with si-NC + Rapamycin and si-NDUFA4L2 groups, the trend is more obvious in si-NDUFA4L2 + Rapamycin groups. Thus, these results confirmed that Rapamycin promoted autophagy. Subsequently, we found that treatment with rapamycin alone did not significantly change E-cadherin and Vimentin protein expression. However, Rapamycin was capable of enhancing the protein-based expression of E-cadherin and was capable of reducing Vimentin expression after silencing of NDUFA4L2 (Figure 4A). Notably, immunofluorescence confirmed all of our above findings (Figure 4B and Supplementary Figures S3F,G). Subsequent to these findings, we next investigated the role of autophagy in the invasion of OS cells transfected with si-NDUFA4L2 by using transwell assays. Figure 4C indicated that autophagy also enhanced the invasion of OS cells transfected with si-NDUFA4L2. These results indicated that autophagy promoted the progression of EMT and invasion of OS cells under a hypoxic environment when NDUFA4L2 was repressed.

To confirm the role of autophagy in migration and proliferation, we used wound healing analysis and colonizing assays for OS cells treated with Rapamycin after transfected with si-NDUFA4L2. As was demonstrated in Supplementary Figure S4A, the migration of HOS cells was repressed, and autophagy was capable of promoting the migration of HOS transfected with si-NDUFA4L2. Furthermore, the results from colony formation assays demonstrated that autophagy could enhance the proliferation of OScells post-transfection with si-NDUFA4L2 (Supplementary Figure S4B). However, treatments with Rapamycin alone did not significantly change the migration and cloning ability of OS cells (Supplementary Figures S4A,B). To further assess whether or not autophagy regulated metastasis and epithelial–mesenchymal transition of osteosarcoma cells though the removal of ROS production, we assessed ROS levels by using Reactive Oxygen Species Assay Kits. The corresponding results demonstrated that Rapamycin reduced ROS levels in OS cells transfected with si-NDUFA4L2 and treatments with Rapamycin alone could also decrease the ROS levels in hypoxic environments (Supplementary Figure S4C). These results indicated that treatments with Rapamycin alone were able to reduce the ROS levels while they did not significantly promote the invasion, migration, proliferation and EMT ability of OS cells. Rapamycin could significantly promote the invasion, migration, proliferation and EMT ability of OS cells transfected with si-NDUFA4L2 in hypoxic environments.

**FIGURE 5:**
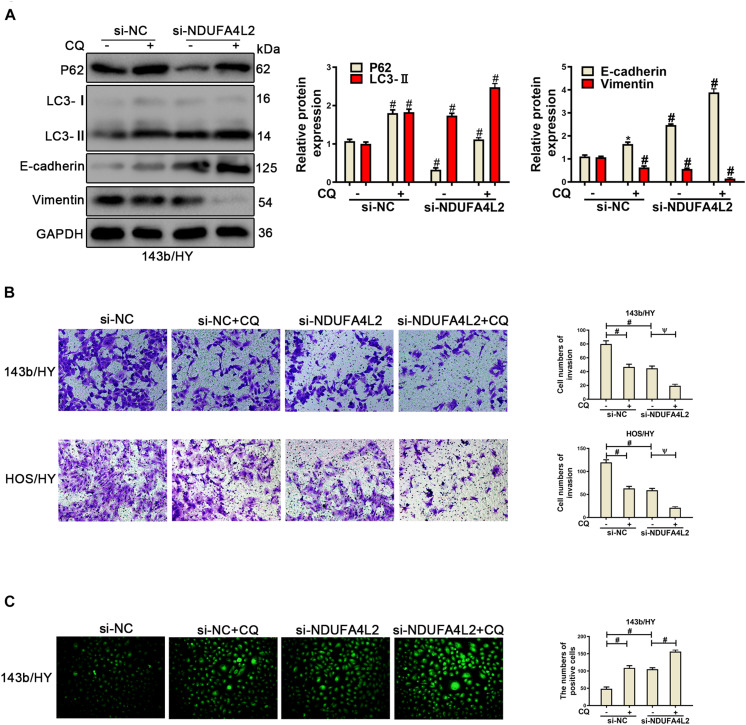
Repression of autophagy could repress the EMT progression of si-NDUFA4L2-transfected 143b and HOS cells. 143b and HOS cells were treated with CQ after transfecting with si-NC or si-NDUFA4L2 in hypoxic environments. **(A)** Western blotting was performed to determine the protein expression of P62, LC3, E-cadherin, Vimentin, and GAPDH in 143b cells. For P62, LC3, E-cadherin, and Vimentin, si-NC vs. si-NC + Rapamycin was # < 0.001, si-NC vs. si-NDUFA4L2 was # < 0.001, si-NDUFA4L2 vs. si-NDUFA4L2 + Rapamycin was # < 0.001. **(B)** Transwell assays were performed to determine the invasion ability of si-NDUFA4L2-transfected 143b and HOS cells after treatment with CQ. **(C)** ROS production was detected by using Reactive Oxygen Detection Kits. HY, hypoxic environment. nsp ≥ 0.05, ψ < 0.01, and # < 0.001 were defined as measures that indicated significant differences among treatment groups. All experiments were performed in triplicate.

To further verify the role of autophagy in OS cells under hypoxic environments when NDUFA4L2 was inhibited, Chloroquine (CQ, 10 μM) (He et al., 2019) was used to treat OS cells transfected with si-NDUFA4L2. As was demonstrated in Figure 5A, CQ caused an increased level of P62 and LC3, which showed autophagy was inhibited, and was likely repressed by CQ. Next, Western blotting and transwell assays were used to assess the effect of autophagy in OS cells. Figure 5A indicated that EMT progression in OS cells were decreased due to the knockdown of NDUFA4L2, and furthermore suggested that CQ enhanced this effect. Additionally, Figure 5B demonstrated that restraint of autophagy further inhibited the invasion of OS cells induced by knockdown of NDUFA4L2. Interestingly, ROS production in OS cells increased (Figure 5C). Importantly, treatments with CQ alone were able to remarkably inhibit EMT and invasion of OS cells as well as increasing ROS levels in hypoxic environments (Figures 5A–C). These results revealed that autophagy promoted EMT progression, invasion, migration, and proliferation in OS cells transfected with si-NDUFA4L2 by eliminating ROS production.

### NDUFA4L2 Knockdown Inhibits Osteosarcoma Growth *in vivo*

**FIGURE 6:**
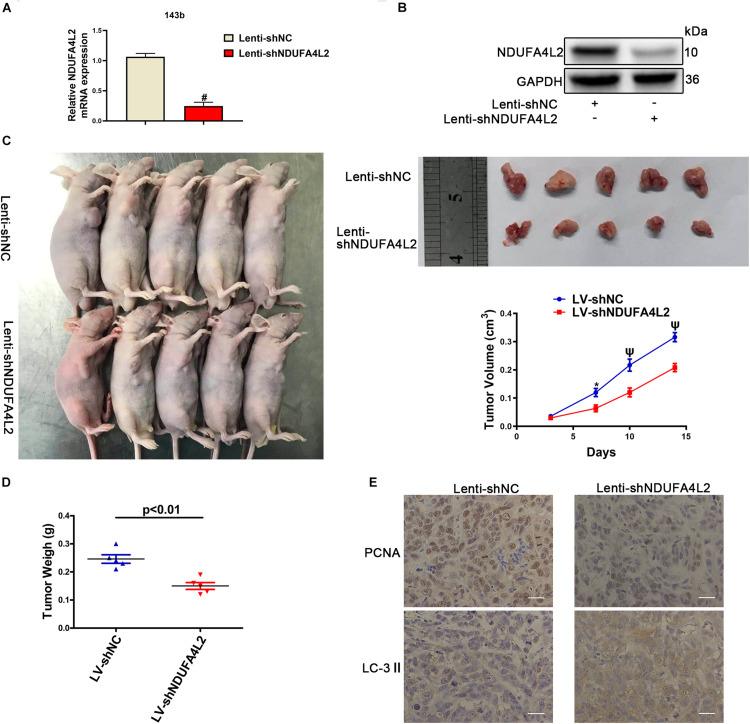
NDUFA4L2 knockdown repressed OS tumor growth *in vivo*. **(A)** The levels of expression of NDUFA4L2 mRNA were detected by using qRT-PCR (*n* = 5). **(B)** NDUFA4L2 protein expression was determined by Western blotting (*n* = 5). **(C)** Results for nude mice carrying tumors from 143b/LV-shNDUFA4L2 and 143b/LV-shNC groups were characterized. Tumor growth curves were assessed weekly (*n* = 5). **(D)** Tumor weight from 143b/LV-shNDUFA4L2 and 143b/LV-shNC groups were characterized (*n* = 5). **(E)** PCNA and LC3 protein expression was determined by using immunohistochemical staining (*n* = 5). nsp ≥ 0.05, ^∗^*p* < 0.05, ψ < 0.01, and # < 0.001 were defined as measures that indicated significant differences among treatment groups.

The function of NDUFA4L2 *in vivo* was evaluated in BALB/c nude mice xenografted with 143b cells. The effect of Lenti-shNDUFA4L2 was confirmed by qRT-PCR and Western blotting. The levels of mRNA and protein expression of NDUFA4L2 in 143b cells were reduced significantly (Figures 6A,B). There was a significant decrease in tumor volume in the Lenti-shNDUFA4L2 treatment group (Figure 6C). It can be seen in Figure 6D that tumor volume was decreased obviously at time steps of 7, 10, and 15 days. Furthermore, tumor weight, PCNA and LC3-*II* were measured. Findings indicated that compared with the Lenti-NC group, tumor weights in the Lenti-NDUFA4L2 group were significantly reduced and expression of PCNA in the Lenti-shNDUFA4L2 group was significantly downregulated in OS tissues derived from nude mice. LC3-*II* protein expression was upregulated (Figure 6E). However, there were no significant differences in HIF-1α expression between Lenti-NC and Lenti-shNDUFA4L2 groups (Supplementary Figure S5A), confirming that NDUFA4L2 knockdown did not change the expression level of HIF-1α and HIF-1α regulate NDUFA4L2 expression. Moreover, HIF-1α was accumulated in the nuclei of cancer cells *in vivo* (Supplementary Figures S5A,B). These results confirmed that knockdown of NDUFA4L2 induced the inhibition of growth of OS tumors and that autophagy could be induced when mitochondrial NDUFA4L2 was silenced.

To confirm the role of NAC in OS tumors transfected with Lenti-shNDUFA4L2, we treated BALB/c nude mice with NAC (7 mg/mL). We found that NAC facilitated the growth of OS tumors (Supplementary Figure S5C). NAC increased tumor volume and weight in the Lenti-shNDUFA4L2 + NAC treatment group (Supplementary Figures S5D,E). PCNA expression increased and LC-3 expression decreased significantly in the Lenti-shNDUFA4L2 + NAC treatment group (Supplementary Figure S5F). These results suggested that NAC could rescue the growth of OS tumors post-knockdown of NDUFA4L2.

## Discussion

Osteosarcoma is one of the most common primary malignant bone tumors that occurs in children, teenagers, and young adults. Typical characteristics of OS are pain and swelling in the affected bone in the place of onset. OS patients often wake from sleep with an intensive pain, which is a hallmark of OS (Cai et al., 2019). Chemotherapy is the most efficient supportive therapy for the treatment of OS (Bielack et al., 2002). However, a large number of patients are prone to developing chemoresistance, which might relate to relatively low levels of improvement of respective 5-year survival rates despite advancements in methodology (Ferrari and Serra, 2015). Therefore, there has been an ongoing need for novel methods that can help to better overcome the limitations of chemotherapy. Accordingly, in our study we firstly confirmed that HIF-1α and NDUFA4L2 were overexpressed in OS cells under hypoxic environments. The ROS production and lactate production was increased in hypoxic environments. OCR was decreased in hypoxic environments. We also found that HIF-1α regulated NDUFA4L2 expression through the HRE (Hypoxic reaction element) in the NDUFA4L2 promoter region in OS cell lines cultured in hypoxic environments. HIF-2α did not regulate NDUFA4L2 expression. High levels of expression of NDUFA4L2 were significantly correlated with apoptosis, cell migration, invasion and EMT progression of OS. In 143b and U2OS, we found that knockdown of NDUFA4L2 inhibited OS cell proliferation, invasion, and migration, as well as induced cell apoptosis through the results based upon functional assays. *In vivo*, we found that NDUFA4L2 knockdown could inhibit the growth of OS tumors. These results revealed that NDUFA4L2 induced by HIF-1α improved the survival, metastasis, and EMT progression of OS cells.

Prior findings have indicated that NDUFA4L2 is upregulated in many kinds of tumors and plays an important role in the hypoxic environments (Tello et al., 2011; Yamamoto and Tsuchiya, 2013; Lai et al., 2016; Wang et al., 2017; Piltti et al., 2018). However, such examinations as related to NDUFA4L2 have yet to be reported upon with respect to OS. In our study, we discovered that NDUFA4L2, a component of the ETC complex I, was upregulated in OS in hypoxic environments. Complex I, which is a key component in the first step of ETC, can transfer electrons from NADPH to a non-covalently bound flavin mononucleotide. In the process of the ubiquinone reduction in complex I and the outer quinone-binding site of the Q cycle in complex III, it has significant ROS production (Raha and Robinson, 2000; Liu et al., 2002; Lv et al., 2017). ROS production mediates homeostasis of redox that correspondingly plays an important role in the survival of cancer cells. In this study, we showed that mitochondrial NDUFA4L2, a component of the ETC complex I subunit, was overexpressed in OS cell lines cultured in hypoxic conditions. OCR was reduced significantly, and the ROS levels was increased in OS cell lines under hypoxic environment. NDUFA4L2 knockdown caused an increased level of ROS and OCR in OS cells under hypoxic environments. These results confirmed that NDUFA4L2 restricted the ETC activity and NDUFA4L2 knockdown increased ROS production and OCR in OS cells through promoting the ETC activity. Based upon our above findings, we inferred that NDUFA4L2 protected OS cell lines from hypoxic environments by facilitating the regulation of redox homeostasis. To test and confirm our hypothesis, the ROS scavenger NAC was applied to OS cells with the effect of silencing of NDUFA4L2. Interestingly, we discovered that NAC can reverse functions of si-NDUFA4L2 to OS cells. However, NAC did not affect the control cells regardless of whether OS cells were cultured in normal or hypoxic environments (Supplementary Figure S3A). From there we evaluated the effects of NAC upon OS cells cultured in normal environments and found that NDUFA4L2 knockdown and NAC did not affect OS cells (Supplementary Figure S3B). NDUFA4L2 was downregulated in OS cells cultured in normoxia environments. NDUFA4L2 knockdown did not cause an increase of ROS production and thereby NAC did not affect OS cells cultured in normoxic environments. Overexpression of NDUFA4L2 in normoxia could not activate the EMT progression. *In vivo*, ROS scavenger NAC could promote the growth of OS tumors although NDUFA4L2 was silenced. These results provided support and lent confirmation to our hypothesis that NDUFA4L2 could promote the survival, metastasis, and EMT progression by inhibition of ROS.

It is well-known that autophagy can mediate apoptosis activity. However, the specific mechanism remains unknown. As a double-edged sword, autophagy could either heighten or repress tumor cell proliferation, invasion, and migration, depending upon the conditions of the environment of the cell and stimuli present (St-Pierre et al., 2002). For osteosarcoma, research has contrastingly both observed that autophagy may either promote or inhibit proliferation under the context of different gene regulation (Kenific et al., 2010; Bao et al., 2018; Zhao et al., 2018). Literature has also indicated that autophagy could enhance cellular activity through anti-oxidative stress (Zhang et al., 2018) and that cells tend to increase oxidative stress under hypoxic conditions (Liang et al., 2015). Herein, we found that autophagy related proteins were overexpressed when 143b and U2OS cells were cultured in hypoxic environments. When using small interference to knock down NDUFA4L2, we observed that autophagy flux was upregulated. Treatments with Rapamycin did not enhance the EMT process, invasion, proliferation and migration of OS cells while could reduce ROS levels in hypoxic environments. These results showed that further autophagy could not enhance the tumor phenotype of OS but could compensate for tumor suppression induced by NDUFA4L2 deletion via reducing ROS levels. However, inhibition of autophagy alone could inhibit the tumor phenotype of OS in hypoxic environments and increase the ROS levels. Inhibition of autophagy may cause an increased level of ROS to inhibit the tumor phenotype of OS. Rapamycin promoted OS cell metastasis and EMT progression *in vitro* and CQ enhanced the effect of si-NDUFA4L2 on OS cells when NDUFA4L2 was knocked down. These findings thus revealed that autophagy was able to compensate for the loss of NDUFA4L2 function with respect to OS cell proliferation, invasion, migration and EMT progression. Silencing of NDUFA4L2 was able to induce autophagy to facilitate rescue of proliferation, invasion, migration, and EMT progression.

There is a shortcoming in this study. The further mechanism of NDUFA4L2 in tumor growth has not been fully demonstrated. In future, we hope that our peers will study this issue with us.

## Conclusion

In conclusion, this study illustrated how the molecular relationship between HIF-1α, NDUFA4L2, oxidative stress, and autophagy mediated the regulation of survival, metastasis and EMT progression of OS cells in hypoxic environments. In hypoxic environments, we found that low oxygen tension induced HIF-1α, such as to promote NDUFA4L2 expression. NDUFA4L2 improved survival, metastasis, and EMT progression of OS cells that survived in hypoxic conditions by facilitating repression of ROS production. When NDUFA4L2 was silenced, a large amount of ROS activated autophagy flux to facilitate maintenance of survival, metastasis, and EMT progression of OS cells. Therefore, the findings in our study helped to elucidate the survival of Osteosarcoma and provided a novel therapeutic target for Osteosarcoma. Further studies are warranted to confirm the clinical significance of NDUFA4L2 and autophagy in Osteosarcoma.

## Data Availability Statement

All datasets generated for this study are included in the article/Supplementary Material.

## Ethics Statement

The animal study was reviewed and approved by the Xinhua Hospital, Shanghai Jiao Tong University School of Medicine.

## Author Contributions

W-NX, S-DJ, and L-SJ conceived and designed the experiments. W-NX and H-LZ performed the experiments. W-NX and R-ZY acquired and analyzed the data. W-NX drafted the manuscript. S-DJ and L-SJ helped perform the analysis with constructive discussions and revised the manuscript. All authors contributed to the article and approved the submitted version.

## Conflict of Interest

The authors declare that the research was conducted in the absence of any commercial or financial relationships that could be construed as a potential conflict of interest.
