# Supplementary material for: NDUFA4L2 Regulated by HIF-1α Promotes Metastasis and Epithelial–Mesenchymal Transition of Osteosarcoma Cells Through Inhibiting ROS Production
Source: Front Cell Dev Biol. 2020 Nov 20;8:515051. doi: 10.3389/fcell.2020.515051 (PMC7714780; doi:10.3389/fcell.2020.515051)
Supplement: Supplementary file 1 [file Data_Sheet_1.docx]

**Figure S1 HIF-1α knockdown decreased the expression of NDUFA4L2 and the bindings to NDUFA4L2’ HRE.** (A) 143b and HOS cells were cultured in hypoxic environments for 24h and 48h. Western blotting was used to detect the protein level of Cytochrome-c in cytoplasm or mitochondria of 143b and HOS cells. (B) Oxygen consumption rates were detected by high-resolution respirometry. (C) lactate production was detected by lactic acid assay kit. (D) A schematic representation of the human NDUFA4L2 gene and nucleotide sequences matching HRE from six mammalian genes is presented. (E) Cell growth rate were detected by CCK-8 kit. (F) Flow cytometry assays were used to facilitate detection of OS cell apoptosis in hypoxic environments after pretreatment with staurosporine. (G-H) Western blotting was used to determine protein levels of HIF-1α, HIF-2α, and NDUFA4L2 in 143b cells cultured in hypoxic environments after HIF-1α and HIF-2α were knocked-down respectively. (I) CHIP assays for assessment of HIF-1α binding to the NDUFA4L2 genes in 143b cells cultured in hypoxic environments after HIF-1α knockdown. (J) CHIP assays of HIF-1α binding to the NDUFA4L2 gene in 143b cells after knockdown of HIF-2α in hypoxic environments. Cyt-c: Cytoplasm cytochrome c; Mit-c: Mitochondrial cytochrome c; HY: hypoxic environment. nsp ≥ 0.05, *p < 0.05, ψ < 0.01, and # < 0.001 were defined as measures that indicated significant differences among treatment groups. All experiments were performed in triplicate.

**Figure S2 Silencing of NDUFA4L2 reduced the migration and promote the apoptosis of OS cells.** (A)143b, U2OS and HOS cells were transfected with si-NC, si-NDUFA4L2-1, si-NDUFA4L2-2 or si-NDUFA4L2-3. Western blotting was used to determine the protein levels of NDUFA4L2 in 143b, U2OS and HOS cells. (B) Relative cell proliferation of U2OS, and HOS cells was detected by CCK-8. (C) Flow cytometry assays were used to detect OS cell apoptosis in hypoxic environments. (D) TUNEL assays were used to detect OS cell apoptosis. (E) Quantitative analysis for immunofluorescence of E-cadherin and Vimentin. (F) The migration of 143b and HOS cells culture in hypoxic environments post-transfection of si-NC or si-NDUFA4L2 was determined by using Wound healing assays. HY: hypoxic environment. nsp ≥ 0.05, *p < 0.05, ψ < 0.01, and # < 0.001 were defined as measures that indicated significant differences among treatment groups. All experiments were performed in triplicate.

**Figure S3 NDUFA4L2 knockdown and NAC do not affect the survival of OS cells under normoxic environments.** (A) Western blotting was used to detect protein levels of Cleaved-caspase 3 and GAPDH in 143b cells treated with NAC in hypoxic environments (C) Western blotting was used to detect protein levels of NDUFA4L2, Cleaved-caspase 3, and GAPDH in 143b cells treated with NAC post-transfection with si-NC or si-NDUFA4L2-1 in normoxia environments. (D) TUNEL assays were used to detect 143b cell apoptosis when 143b cells had been treated with NAC and post-transfection with si-NC or si-NDUFA4L2-1 in hypoxic environments. (E) Western blotting was used to detect protein expression of E-cadherin and Vimentin in 143b cells, U2OS and HOS cells treated with NAC post-transfection with si-NC or si-NDUFA4L2-1 in hypoxic environments. (F-G) Quantitative analysis for immunofluorescence of E-cadherin and Vimentin. NX: normoxic environment; HY: hypoxic environment. nsp ≥ 0.05, *p < 0.05, ψ < 0.01, and # < 0.001 were defined as measures that indicated significant differences among treatment groups. All experiments were performed in triplicate.

**Figure S4 Upregulation of autophagy promoted migration and proliferation of si-NDUFA4L2-transfected 143b, U2OS and HOS.** 143b, U2OS, and HOS cells were treated with Rapamycin post-transfection with si-NC or si-NDUFA4L2. (A) Wound healing analyses were used to determine the colonizing ability of si-NDUFA4L2-transfected HOS cells post-treatment with Rapamycin. (B) Colony formation assays were performed to determine the colonizing ability of si-NDUFA4L2-transfected 143b, U2OS, and HOS cells post-treatment with Rapamycin. (C) ROS production in 143b cells was detected by using a Reactive Oxygen Detection Kit. nsp ≥ 0.05, *p < 0.05, ψ < 0.01, and # < 0.001 were defined as measures that indicated significant differences among treatment groups. All experiments were performed in triplicate.

**Figure S5 HIF-1α protein expression was not significantly different between Lenti-NC group and Lenti-shNDUFA4L2 group.** (A) HIF-1α protein expression was determined by immunohistochemical staining (n=5). (B) Western blotting was used to determine HIF-1α protein level in cytosol and nuclear of OS cell in vivo (n=3). (C) Results for nude mice carrying tumors from 143b/LV-shNDUFA4L2 + NAC, 143b/LV-shNDUFA4L2 and 143b/LV-shNC groups were characterized (n=5). (D) Tumor growth curves were assessed weekly (n=5). (E) Tumor weight from 143b/LV-shNDUFA4L2 + NAC, 143b/LV-shNDUFA4L2 and 143b/LV-shNC groups were characterized (n=5). (F) PCNA and LC3 protein expression was determined by using immunohistochemical staining (n=5). *p < 0.05 and **< 0.01 were defined as measures that indicated significant differences among treatment groups. All experiments were performed in triplicate.
