# Supplementary figures and images for: NDUFA4L2 Regulated by HIF-1α Promotes Metastasis and Epithelial–Mesenchymal Transition of Osteosarcoma Cells Through Inhibiting ROS Production
Source: Front Cell Dev Biol. 2020 Nov 20;8:515051. doi: 10.3389/fcell.2020.515051 (PMC7714780; doi:10.3389/fcell.2020.515051)

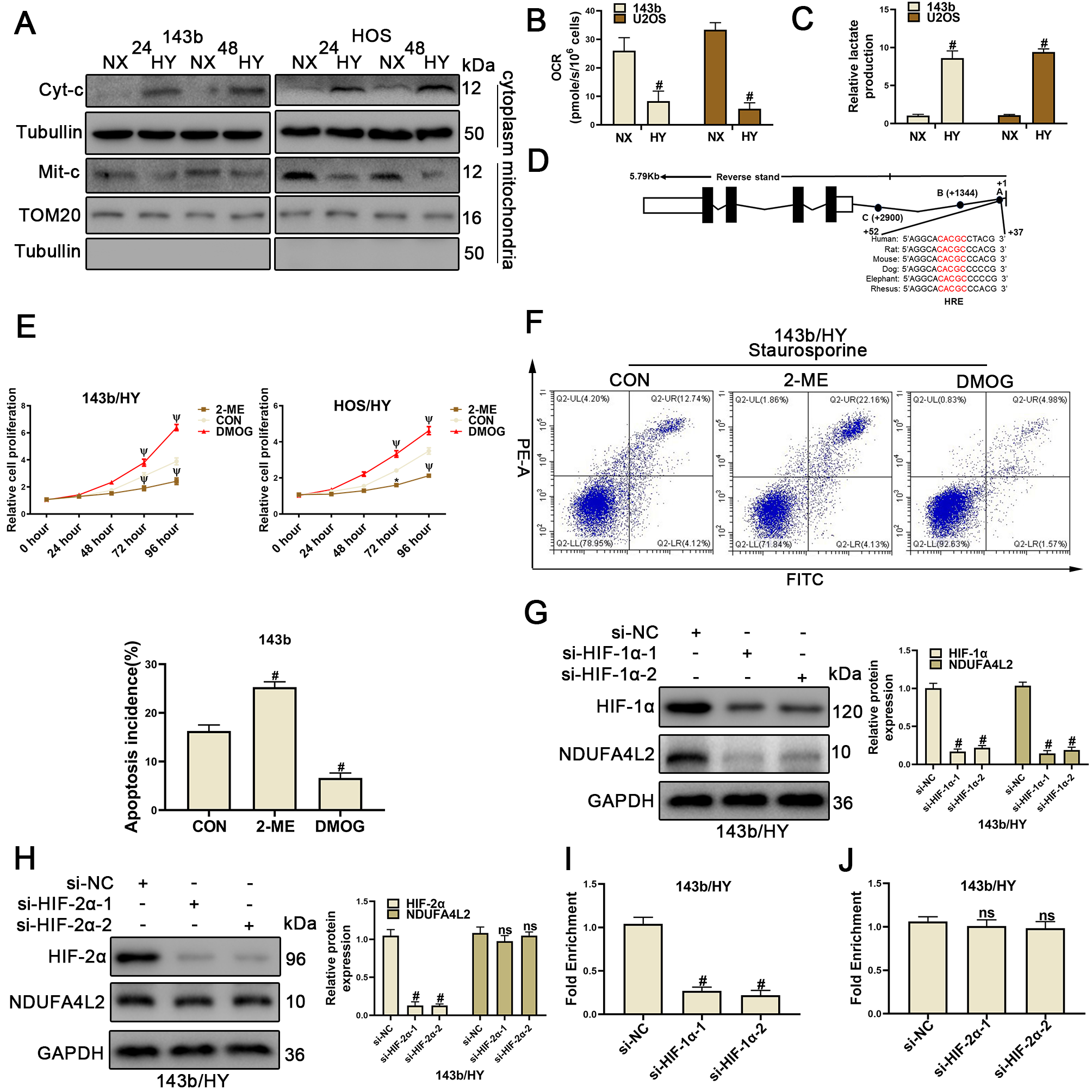

Supplement: Supplementary file 2 [file Image_1.TIF]

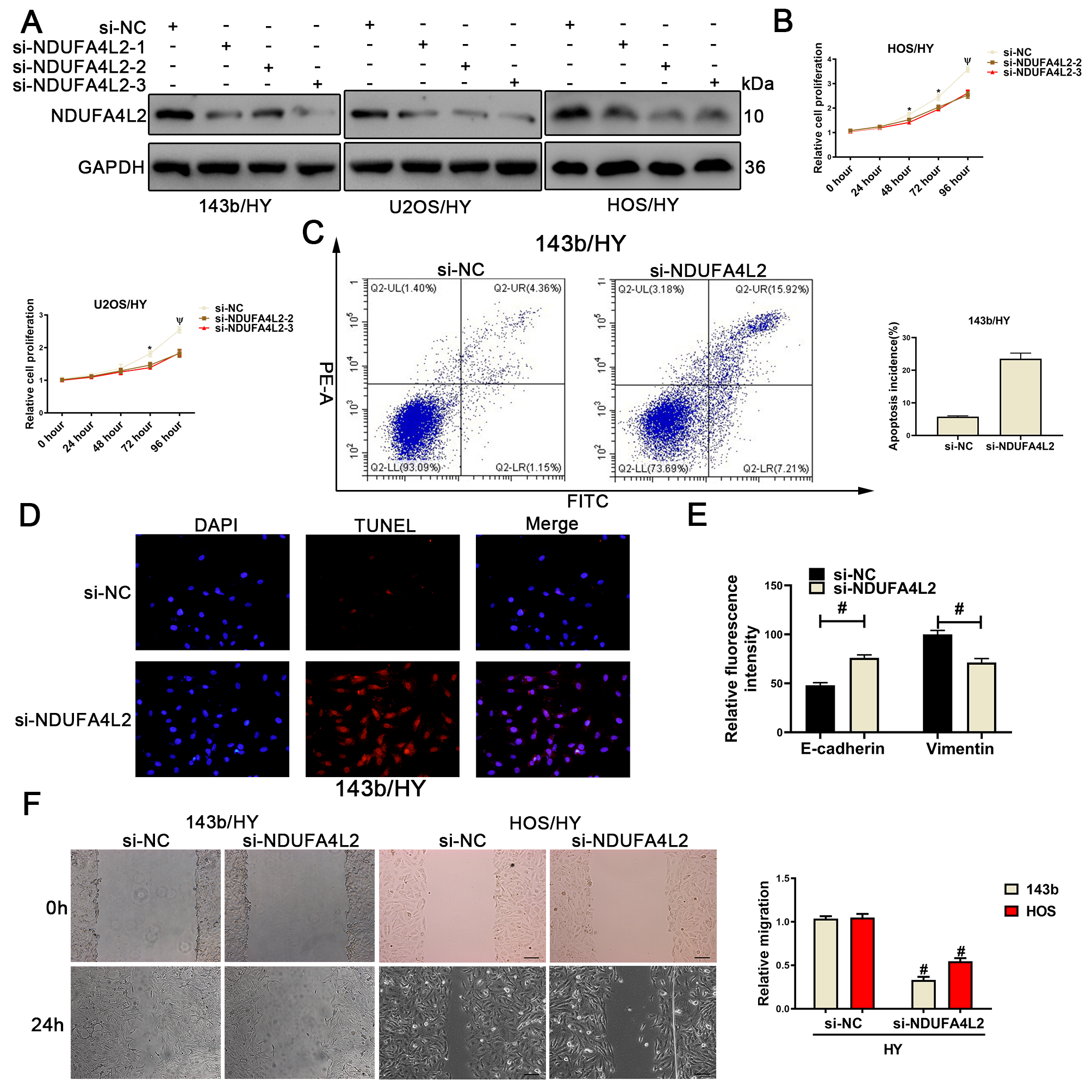

Supplement: Supplementary file 3 [file Image_2.TIF]

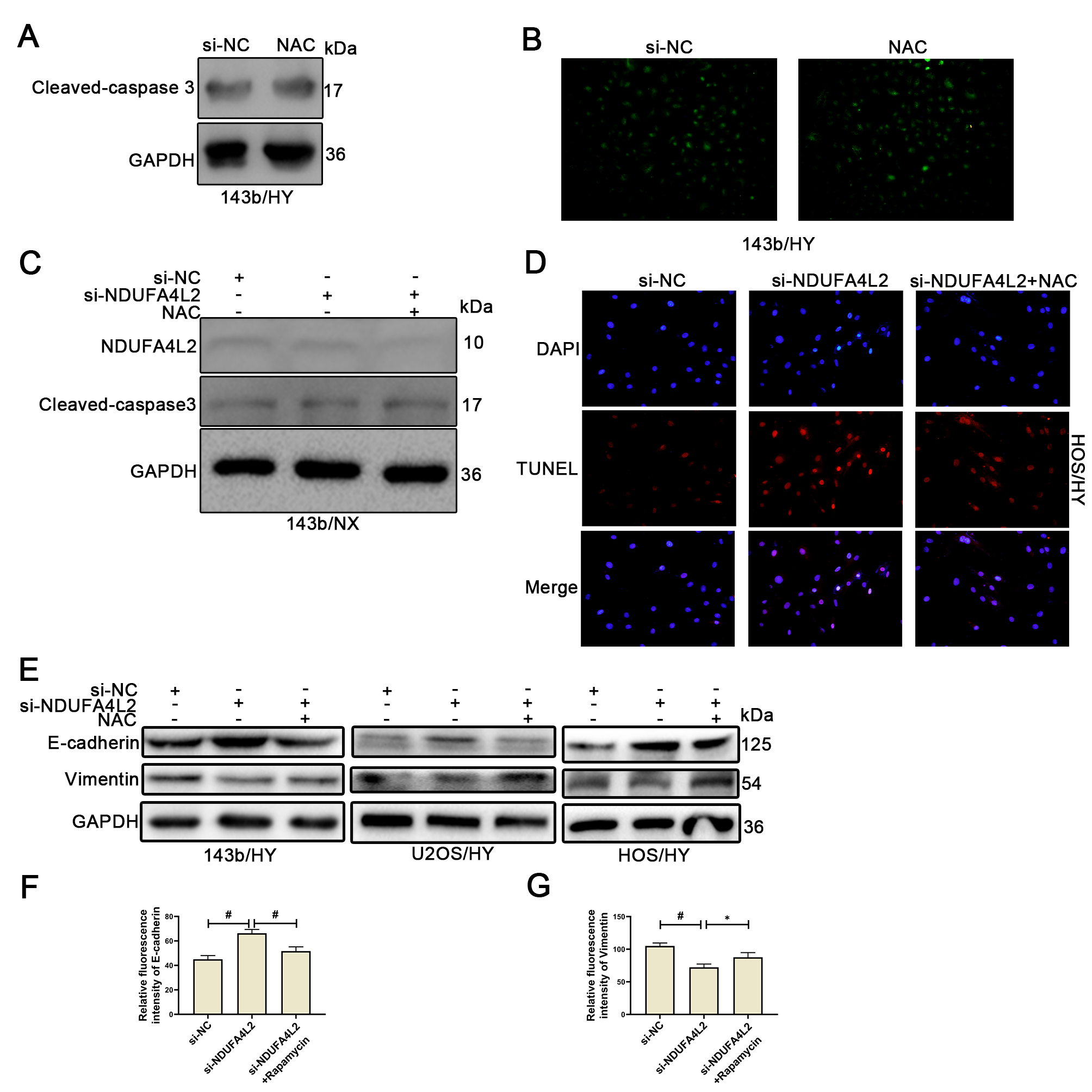

Supplement: Supplementary file 4 [file Image_3.TIF]

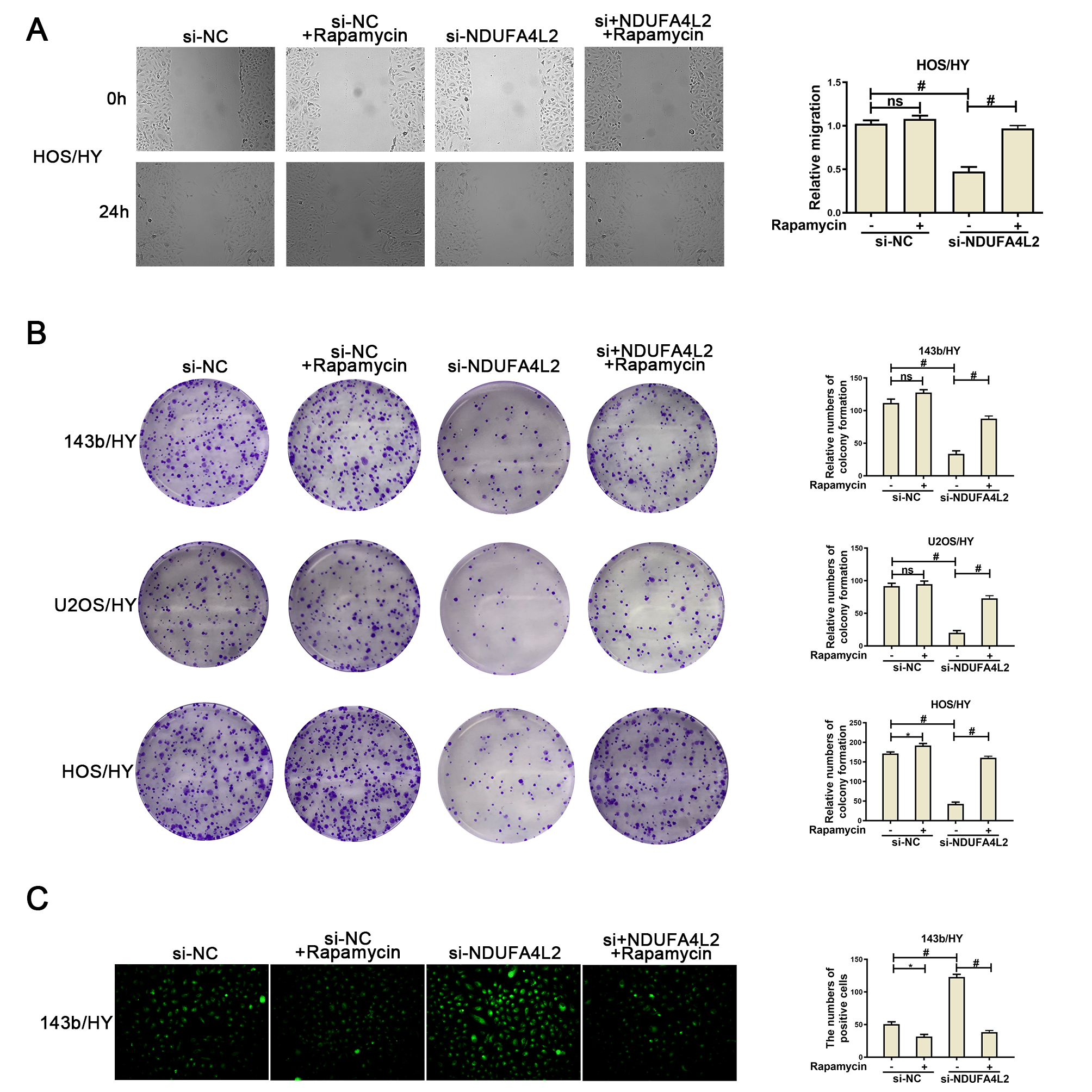

Supplement: Supplementary file 5 [file Image_4.TIF]

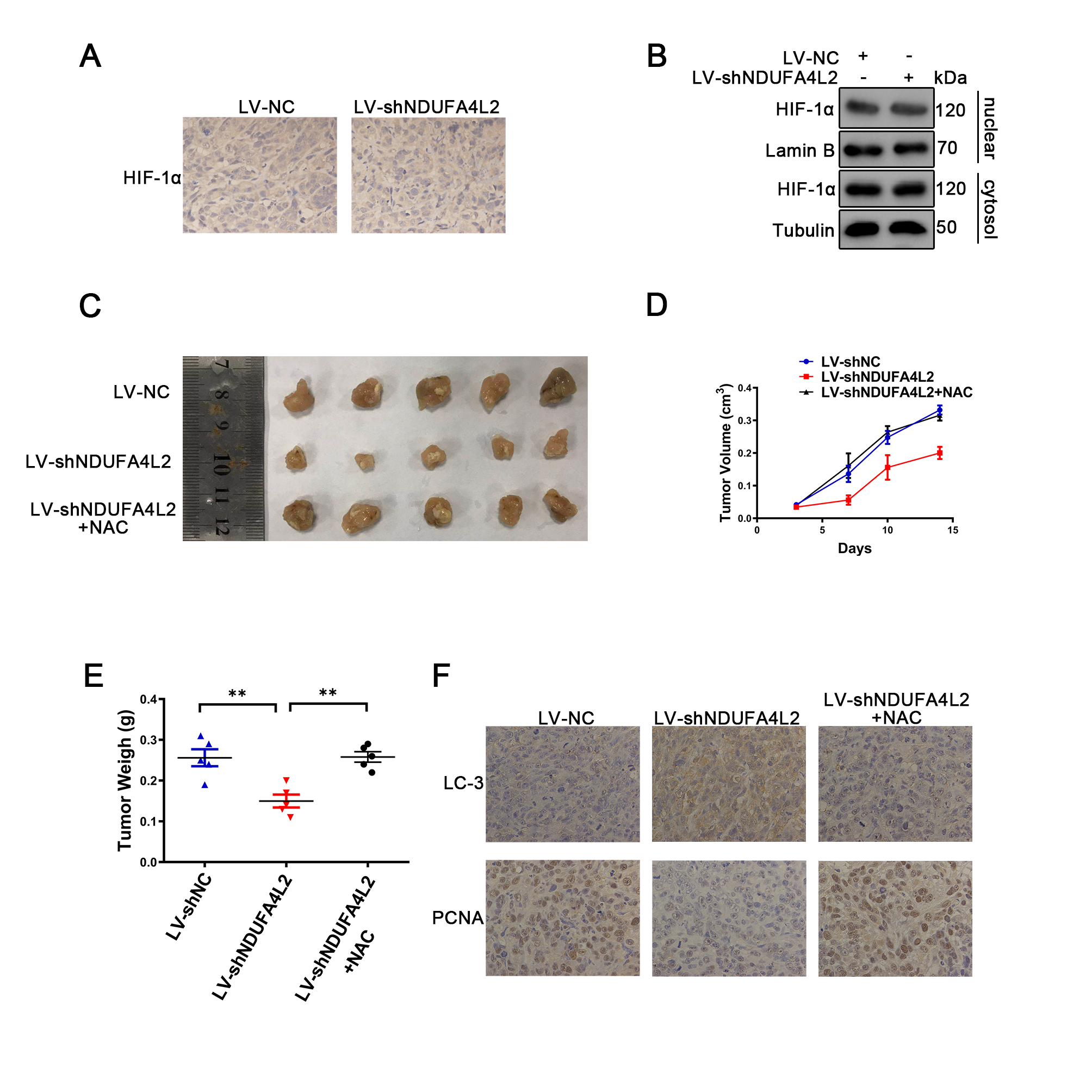

Supplement: Supplementary file 6 [file Image_5.TIF]
